# Accuracy, Relevance, and Redundancy of ChatGPT-5.3 References in Open Fracture Management: A Methodological Analysis

**DOI:** 10.7759/cureus.116893

**Published:** 2026-09-25

**Authors:** Gündüz Ercan Kutluay, Yusuf Kondil

**Affiliations:** 1 Orthopedics and Traumatology, ÇOSB Kapaklı State Hospital, Tekirdağ, TUR; 2 Orthopedics and Traumatology, Tekirdağ Namık Kemal University, Tekirdağ, TUR

**Keywords:** artificial intelligence, bibliographic accuracy, chatgpt, open fracture, orthopedic trauma, reference accuracy

## Abstract

Objective: This study aimed to evaluate the bibliographic accuracy of the references that ChatGPT-5.3 (OpenAI, San Francisco, CA) presented as PubMed-indexed sources for open fracture management and determine whether the verifiable references were relevant to the topics they were meant to support.

Methodology: In a single session in March 2026, ChatGPT-5.3 (Plus subscription), with the web search function enabled, was asked to systematically describe the management of open fractures. The model was then prompted to provide three PubMed-indexed references for each topic it had generated. ChatGPT-5.3 structured open fracture management into 15 topic categories and produced a total of 45 references. Two independent reviewers assessed each reference for bibliographic accuracy and content relevance. Bibliographic accuracy was scored from 0 to 2, and content relevance from 0 to 1. Final scores were determined by consensus. Inter-rater agreement was assessed using Cohen’s kappa coefficient.

Results: Of the 45 references, 11 (24.4%) were fully bibliographically accurate. Nineteen references (42.2%) existed but contained errors in the PMID, year, journal, title, or other bibliographic details. Fifteen references (33.3%) could not be verified as valid PubMed-indexed articles. The overall bibliographic accuracy rate was 11 (45.6%), the content relevance rate was 30 (66.7%), and the overall performance score was 52.6%. All bibliographically verifiable references were content-relevant to their assigned open fracture management topics, with 30 (66.7%) references being content-relevant. The 45 references corresponded to only 36 unique sources; a single review article was reproduced under 8 (53.3%) of the 15 topic categories and accounted for 8 (72.7%) of the 11 fully accurate references. In a sensitivity analysis restricted to unique sources, the bibliographic accuracy rate decreased to 13 (37.5%). Inter-rater agreement was high, with Cohen’s kappa values of 0.966 for bibliographic accuracy and 0.951 for content relevance.

Conclusions: Although ChatGPT-5.3 generated a clinically reasonable and systematic framework for open fracture management, the references provided to support this response showed substantial bibliographic reliability problems. Because all verifiable references were content-relevant, the model’s principal limitation appeared to lie not in the selection of irrelevant sources, but in the presentation of seemingly appropriate sources with inaccurate or unverifiable bibliographic information. References generated by ChatGPT should not be used for academic or clinical purposes without independent verification.

## Introduction

Large language models are increasingly used to access medical information and support academic writing, clinical education, and clinical decision-making. Artificial intelligence (AI)-based conversational models such as ChatGPT can generate rapid, structured, and linguistically coherent responses to complex medical questions and are therefore widely regarded as supportive tools by medical students, residents, clinicians, and researchers [1,2]. In orthopedics and traumatology, the potential applications of these models have also been discussed in the contexts of education, patient information, examination performance, and clinical knowledge generation [3,4].

However, a convincing and systematic presentation of medical content does not, by itself, ensure reliability. Clinical and academic reliability depends on whether the information provided is supported by appropriate and accessible sources. In scientific writing, references are required not only to support claims, but also to allow readers, reviewers, and editors to trace the origin of the information. In medical texts, therefore, the source of information matters as much as the information itself.

One of the major limitations of large language models is hallucination, which refers to the generation of outputs that are formally coherent and convincing but cannot be verified against factual information. In academic writing, this may take the form of nonexistent article titles, incorrect author details, erroneous journal or publication-year information, or PMID numbers belonging to different articles [5]. Previous studies have shown that the use of unverifiable or inaccurate sources may represent an important problem in AI-generated scientific texts [5].

Open fracture management is one of the most critical and time-sensitive areas of orthopedic trauma. It requires a multistep approach that includes initial trauma assessment, limb examination, early antibiotic administration, tetanus prophylaxis, surgical irrigation and debridement, fracture stabilization, soft tissue coverage, vascular injury management, and complication surveillance [6,7]. Each of these components has been addressed in the literature as a distinct area of research and review, and clinical knowledge on open fracture management has been shaped by cumulative evidence from multiple subfields.

Although previous studies have evaluated the bibliographic accuracy and relevance of AI-generated references in various medical fields, evidence remains limited in orthopedic trauma. To our knowledge, no study has specifically assessed ChatGPT-generated PubMed-indexed references for open fracture management by jointly evaluating bibliographic accuracy and topic-specific content relevance. Therefore, this study aimed to assess the bibliographic accuracy of the references generated in a single ChatGPT-5.3 (OpenAI, San Francisco, CA) response to prompts about open fracture management. Among references that could be linked to a verifiable PubMed record, we also assessed whether the underlying source was relevant to its assigned management topic. A secondary aim was to describe reference duplication across topic categories.

## Materials and methods

Study design

This methodological study assessed the bibliographic accuracy and content relevance of the references that ChatGPT-5.3 presented as PubMed-indexed sources for open fracture management in orthopedic trauma. Because no patient data, clinical images, laboratory results, or personal health information were used, the study did not constitute human participant research requiring ethics committee approval.

The study aimed to determine not only whether the references generated by ChatGPT-5.3 were bibliographically real and verifiable, but also, among those that could be linked to a verifiable record, whether they supported the relevant orthopedic topic in terms of content. The evaluation was therefore conducted along two dimensions: bibliographic accuracy and content relevance. Because large language model outputs are non-deterministic and may vary between sessions, this study was designed as an analysis of a single representative ChatGPT-5.3 output rather than as an estimate of the model’s average performance; the rates reported here therefore characterize this specific output.

AI query process

The ChatGPT-5.3 queries were performed in March 2026, shortly after the model’s release, using a ChatGPT Plus subscription with the web search function enabled. All queries were carried out in a single session, and each response was generated once. ChatGPT-5.3 was first asked the following question to systematically describe open fracture management in orthopedic trauma: “What is the management approach for open fractures in orthopedic trauma? Please describe the key steps systematically.”

In response to this query, ChatGPT-5.3 presented open fracture management as a structured framework under 15 main categories. These categories included the key components of open fracture management, such as initial trauma assessment, limb assessment, wound coverage and temporary immobilization, early antibiotic administration, antibiotic selection, tetanus prophylaxis, imaging, Gustilo-Anderson classification, irrigation and debridement, fracture stabilization, soft tissue coverage, negative pressure wound therapy, vascular injury management, compartment syndrome surveillance, and complication follow-up. The complete original response is provided in Appendices A-C. For presentation in Table 1, the AI-generated content was condensed and formatted by the authors without altering its underlying clinical meaning.

In the same session, ChatGPT-5.3 was then prompted to provide PubMed-indexed references supporting each category using the following follow-up question: “Please provide PubMed-indexed references to support each of the steps mentioned above. For every topic give three citations.”

In response to this second query, ChatGPT-5.3 generated three references for each topic category, yielding a total of 45 references. All generated references were recorded without correction and used as a separate dataset for evaluation. The complete prompt-response sequence and all 45 references exactly as generated by the model are provided in Appendices A-C.

Reference assessment

The 45 references generated by ChatGPT-5.3 were reviewed by two independent reviewers. Each reference was first assessed for bibliographic accuracy and then for content relevance. For bibliographic verification, the title, author information, journal name, publication year, volume/issue/page details, and PMID number of each reference were compared with PubMed records. In addition, whether the provided PMID actually corresponded to the cited article was checked. No restriction was placed on the sources consulted during verification; PubMed served as the primary database, and any accessible academic database could be used to confirm or refute a citation.

The references were evaluated exactly as generated by ChatGPT-5.3; no corrections were made before assessment. An incorrect PMID, wrong publication year, incorrect journal name, incomplete or inaccurate title, incomplete author information, or a PMID belonging to a completely different article was considered a bibliographic error. References that could not be verified as PubMed-indexed articles, sources that were books or guidelines rather than PubMed-indexed articles, and sources that could not be verified through online academic databases were classified as bibliographically unverifiable references.

For bibliographically verifiable references, the article content was further evaluated to determine whether it supported the open fracture management topic assigned by ChatGPT. This assessment was based on the direct relevance of the article to the corresponding topic category. For example, references listed under the antibiotic category were examined to determine whether they supported antibiotic prophylaxis, antibiotic timing, or infection prevention strategies in open fractures; references listed under the soft tissue coverage category were assessed for their relevance to flap coverage, early coverage, or orthoplastic management. Detailed reference-level explanations supporting the final consensus classifications are provided in Appendix D.

Because the model was free to reproduce the same source under more than one topic category, the 45 generated references were additionally examined for redundancy. Two references were treated as duplicates when they referred to the same PubMed record, irrespective of the topic category under which they were generated. Duplicate detection was performed independently by both reviewers and confirmed by consensus.

Scoring system

The three-level bibliographic accuracy scale follows the grading approach previously applied by Ghanem et al. to ChatGPT-generated references in trauma surgery, in which references were graded as nonexistent, inaccurate, or accurate [8]. The present study extends that framework by adding a separate content relevance dimension and by applying the assessment across 15 subdomains of a single multistep clinical process.

Each reference was evaluated using two separate scores: bibliographic accuracy and content relevance.

Bibliographic accuracy was scored from 0 to 2:

0/2: The reference could not be verified as a valid PubMed-indexed article, did not exist, represented a non-article source such as a book or guideline, or could not be accessed and verified.

1/2: The reference existed but contained a clear error in the PMID, year, journal, title, author information, or other bibliographic details and required correction.

2/2: The reference was fully consistent with the PubMed record and required no correction.

Content relevance was scored from 0 to 1:

0/1: The article content did not support the assigned open fracture management topic, or content relevance could not be assessed because the reference was not bibliographically verifiable.

1/1: The article content supported the assigned open fracture management topic.

In distinguishing 1/2 from 0/2, a reference was scored 1/2 when a PubMed article matching the cited title, authors, and journal could be identified despite an incorrect identifier or other bibliographic error. It was scored 0/2 when no such article could be identified, or when the source was not a journal article (e.g., a textbook or guideline).

Accordingly, the maximum total score for each reference was 3. The bibliographic accuracy and content relevance scores for each reference were recorded separately in the summary evaluation table.

Inter-rater evaluation process

All references were evaluated separately by two independent reviewers, who were the two authors of the present study and both orthopedic surgery specialists. Each reviewer independently scored all 45 references for bibliographic accuracy and content relevance using the scale defined above. After the independent reviewer scores were recorded, references with discrepant scores were re-examined, and final scores were determined by consensus. The independent reference-level scores assigned by Reviewer 1 and Reviewer 2 are presented in Appendices E and F, respectively, and the final consensus scores are provided in Appendix G.

Inter-rater agreement was assessed separately for bibliographic accuracy and content relevance using Cohen’s kappa coefficient. The final consensus scores were used in the main analyses.

Statistical analysis

Data were analyzed using descriptive statistics. Bibliographic accuracy, content relevance, and total scores were calculated for the 45 references generated by ChatGPT-5.3. Because bibliographic accuracy was scored from 0 to 2 and content relevance from 0 to 1, the maximum total score for each reference was 3.

For bibliographic accuracy, references were classified into three groups: unverifiable references, existing but inaccurate references, and fully accurate references. For content relevance, references were classified as content-relevant or not content-relevant/not assessable.

The overall bibliographic accuracy rate was calculated by dividing the total bibliographic accuracy score by the maximum possible bibliographic accuracy score; this represents a normalized score rather than the proportion of references that were fully accurate. The overall content relevance rate was calculated by dividing the total content relevance score by the maximum possible content relevance score. In addition, an overall performance score was calculated based on the sum of bibliographic accuracy and content relevance scores. This composite expresses the total attained score as a proportion of the maximum attainable score, applying to the combined two-dimensional assessment the normalization approach that Ghanem et al. used for bibliographic accuracy alone [8]. Because the bibliographic accuracy scale has three levels and the content relevance scale two, bibliographic accuracy contributes twice the weight of content relevance within this index, which was therefore treated as a descriptive summary measure rather than as an independent primary outcome.

For each of the 15 open fracture management topic categories, topic-level bibliographic accuracy, content relevance, and total scores were calculated descriptively. No statistical comparisons were performed between topic categories. Inter-rater agreement was assessed separately for bibliographic accuracy and content relevance scores using Cohen’s kappa coefficient.

In addition to the primary analysis, in which each of the 45 generated references was treated as a separate observation, a sensitivity analysis was performed on the set of unique sources. In this analysis, each repeated source contributed a single observation, and the bibliographic accuracy, content relevance, and overall performance rates were recalculated accordingly. Cohen’s kappa coefficient was calculated without weighting, and percent agreement was also reported. A quadratic weighted kappa was additionally calculated for the ordinal bibliographic accuracy scale. Ninety-five percent confidence intervals for the main proportions were calculated using the Wilson method. Statistical analyses were performed using R software version 4.3.0 (R Foundation for Statistical Computing, Vienna, Austria).

## Results

ChatGPT-5.3 generated a total of 45 references for open fracture management in orthopedic trauma, with three references provided under each of the 15 topic categories. The structured open fracture management framework based on the 15 topic categories used in the reference-generation and evaluation process is presented in Table 1. According to the final consensus assessment, 11 (24.4%) of the 45 references were bibliographically fully accurate. Nineteen (42.2%) references existed but contained errors in the PMID, year, journal, title, or other bibliographic details. Fifteen (33.3%) references could not be verified as valid PubMed-indexed articles.

**Table 1:** Systematic open fracture management framework used for reference generation The management domains and key components were generated by ChatGPT-5.3 in response to the initial study prompt and were subsequently condensed and formatted by the authors without altering the underlying clinical meaning. The complete original AI-generated response is provided in Appendices A-C.

No.	Management domain	Key components
1	Initial trauma assessment/ATLS-DCO	Initial management follows general trauma principles: treat the patient first and the fracture second. Airway with cervical spine protection, breathing, circulation with hemorrhage control, disability/neurologic status, and exposure are assessed according to Advanced Trauma Life Support (ATLS) principles. In polytrauma or physiologically unstable patients, damage control orthopedic (DCO) principles are considered before definitive reconstruction.
2	Limb assessment/neurovascular examination	After initial stabilization, the injured limb is evaluated systematically. Wound size, location, contamination, bone exposure, soft tissue loss, and tendon or muscle injury are documented. Distal pulses, capillary refill, skin temperature, motor function, sensory function, and signs of compartment syndrome are assessed and recorded before intervention.
3	Sterile dressing/temporary immobilization	The open wound is promptly covered with a sterile dressing. Gross contaminants may be gently removed or lightly irrigated with sterile saline, but repeated probing or aggressive emergency department washout is avoided. A sterile saline-soaked dressing and sterile bandage are applied, and the limb is immobilized with an appropriate splint. Bedside wound closure is avoided.
4	Early intravenous antibiotics	Intravenous antibiotics are administered as early as possible, ideally within the first hour after presentation. Antibiotic treatment should not be delayed for imaging, operative planning, or definitive classification. The initial regimen is started promptly and later adjusted according to injury severity, contamination type, institutional protocol, and culture results when relevant.
5	Antibiotic selection by Gustilo severity	Antibiotic selection is guided by open fracture severity and contamination pattern. Gustilo type I–II injuries are commonly treated with a first-generation cephalosporin such as cefazolin. Type III injuries may require additional gram-negative coverage. Farm, fecal, or soil contamination may require anaerobic coverage, while water-related injuries require tailored antimicrobial coverage.
6	Tetanus prophylaxis	Tetanus immunization status is assessed in every patient with an open fracture. A tetanus booster is administered when indicated. In patients with incomplete or unknown vaccination status and a contaminated or dirty wound, tetanus immune globulin is considered in addition to vaccination.
7	Imaging	Radiographic evaluation includes anteroposterior and lateral views of the injured segment, ideally including the joint above and below the fracture. Imaging defines fracture morphology, displacement, bone loss, and joint involvement. Computed tomography may be used for suspected intra-articular extension, complex periarticular anatomy, or detailed preoperative planning, but imaging should not delay antibiotics or urgent operative treatment.
8	Gustilo-Anderson classification	The Gustilo-Anderson classification is used for grading open fractures. Type I injuries involve small, relatively clean wounds; type II injuries involve larger wounds with moderate soft tissue damage; and type III injuries involve severe soft tissue injury, contamination, periosteal stripping, or vascular compromise. Type III injuries are further classified as IIIA, IIIB, and IIIC. Definitive classification is most accurate after operative exploration and debridement.
9	Irrigation and surgical debridement	Operative irrigation and debridement are central to open fracture management. The goals are to remove devitalized tissue, foreign material, and contaminants; reduce bacterial burden; and reassess the fracture and soft tissue envelope. The wound may be extended for adequate exposure. Nonviable skin, fascia, muscle, and bone are excised. Repeat debridement may be required when tissue viability is uncertain. The traditional “6-hour rule” is not absolute; urgency depends on contamination, vascular status, compartment syndrome, and injury severity.
10	Fracture stabilization	Stabilization supports pain control, soft tissue recovery, infection control, and rehabilitation. The fixation method is selected according to fracture pattern, soft tissue condition, contamination, patient physiology, and planned reconstruction. Options include temporary splinting, external fixation, plate or intramedullary nail fixation, and staged fixation in damage-control settings.
11	Soft tissue coverage/flaps	Soft tissue management is an integral part of open fracture treatment. The wound is not closed prematurely if contamination, devitalized tissue, or soft tissue uncertainty remains. Coverage options include delayed primary closure, split-thickness skin grafting, local flaps, and free tissue transfer. For severe open fractures, especially Gustilo type IIIB injuries, early coordinated orthoplastic management and timely flap coverage are emphasized.
12	Negative pressure wound therapy	Negative pressure wound therapy may be used as an adjunct in selected open fractures, particularly when immediate definitive closure or flap coverage is not possible. It can temporarily help manage the wound environment between debridement and definitive soft tissue coverage. It is not a substitute for adequate debridement, stable fixation, or timely reconstruction.
13	Vascular injury/ischemic limb	Open fractures with vascular injury require urgent recognition and management. Absent pulses, expanding hematoma, active arterial bleeding, distal ischemia, or neurologic compromise prompt immediate vascular assessment. Initial reduction and splinting may restore perfusion. If ischemia persists, urgent vascular consultation, imaging when appropriate, temporary shunting, and revascularization are considered. Warm ischemia time is critical.
14	Compartment syndrome surveillance	Patients with open fractures remain at risk for acute compartment syndrome even when the wound is open. Pain out of proportion, pain with passive stretch, tense compartments, neurologic symptoms, or deteriorating perfusion should raise concern. Serial clinical examinations are essential. Compartment pressure measurement may be used when uncertain, and fasciotomy should not be delayed if compartment syndrome is suspected.
15	Complications/infection/nonunion follow-up	Open fractures require close follow-up for early and late complications. Early complications include infection, soft tissue necrosis, compartment syndrome, vascular compromise, and thromboembolic events. Late complications include delayed union, nonunion, malunion, chronic osteomyelitis, stiffness, and post-traumatic arthritis. Follow-up assesses wound healing, infection, fixation stability, bone union, alignment, motion, and function.

The total bibliographic accuracy score was 41 out of 90, corresponding to an overall bibliographic accuracy rate of 45.6%; this rate is a normalized score rather than the proportion of accurate references. The proportions of fully accurate, existing but inaccurate, and unverifiable references were 24.4% (95% CI, 14.2%-38.7%), 42.2% (95% CI, 29.0%-56.7%), and 33.3% (95% CI, 21.4%-47.9%), respectively. In terms of content relevance, 30 (66.7%; 95% CI, 52.1%-78.6%) references were relevant to their assigned open fracture management topic. Fifteen (33.3%) references were classified as not content-relevant/not assessable because they could not be bibliographically verified. All 30 bibliographically verifiable references were content-relevant to their assigned topics (30 of 30, 100%). The detailed bibliographic verification and content-relevance assessments are provided in Appendix D, and reference-level consensus scores are presented in Appendix G.

When bibliographic accuracy and content relevance were evaluated together, the total performance score was 71 out of 135. Accordingly, the overall reference performance score for this output was 52.6%. Because content relevance could not be assessed for bibliographically unverifiable references, such references contributed zero to both components of the composite score; the index therefore reflects unverifiability in both components and should not be interpreted as an independent estimate of model performance.

Inter-rater agreement was high. A scoring discrepancy between the two reviewers was identified in only one reference. Percent agreement between the two reviewers was 97.8% (44 of 45) for both dimensions. Cohen’s kappa was 0.966 for bibliographic accuracy and 0.951 for content relevance; the quadratic weighted kappa for the ordinal bibliographic accuracy scale was 0.981. In the pre-consensus cross-tabulation for bibliographic accuracy, the two reviewers jointly assigned 0/2 to 15 references, 1/2 to 18 references, and 2/2 to 11 references, with the single discrepant reference scored 1/2 by Reviewer 1 and 0/2 by Reviewer 2. For content relevance, they jointly assigned 0/1 to 15 references and 1/1 to 29 references, with the same reference accounting for the only disagreement. Because content relevance was assessed only among bibliographically verifiable references, the two categories were unbalanced, and the kappa value for this dimension should therefore be interpreted alongside the raw cross-tabulation rather than in isolation. The single discrepant assessment and its resolution by consensus can be compared across Appendices E-G.

The 15 open fracture management topic categories were evaluated descriptively. Because each topic category included three references, the maximum possible score for each category was 6 for bibliographic accuracy, 3 for content relevance and 9 for the total score. Topic-level total scores ranged from 2/9 to 8/9. The highest total score was observed for early intravenous antibiotic administration: 8/9 (88.9%). The lowest total scores were observed for initial trauma assessment and complications/infection/nonunion follow-up: 2/9 (22.2%). No statistical comparisons were performed between topic categories; these findings are presented descriptively.

The distribution of bibliographic accuracy, content relevance and total scores according to topic category is summarized in Table 2.

**Table 2:** Bibliographic accuracy, content relevance, and total scores according to topic categories. Three references were evaluated within each topic category. Bibliographic accuracy was scored on a scale of 0-2 for each reference, while content relevance was scored on a scale of 0-1. Thus, the maximum possible score for each topic category was 6 for bibliographic accuracy, 3 for content relevance, and 9 for the total score. ATLS, Advanced Trauma Life Support; DCO, Damage Control Orthopedics

No.	Topic category	Bibliographic accuracy score	Content relevance score	Total score
1	Initial trauma assessment/ATLS-DCO	1/6	1/3	2/9
2	Limb assessment/neurovascular examination	2/6	1/3	3/9
3	Sterile dressing/temporary immobilization	2/6	1/3	3/9
4	Early intravenous antibiotics	5/6	3/3	8/9
5	Antibiotic selection by Gustilo severity	4/6	3/3	7/9
6	Tetanus prophylaxis	3/6	2/3	5/9
7	Imaging	2/6	1/3	3/9
8	Gustilo-Anderson classification	4/6	3/3	7/9
9	Irrigation and surgical debridement	4/6	3/3	7/9
10	Fracture stabilization	3/6	2/3	5/9
11	Soft tissue coverage/flaps	2/6	2/3	4/9
12	Negative pressure wound therapy	3/6	3/3	6/9
13	Vascular injury/ischemic limb	3/6	2/3	5/9
14	Compartment syndrome surveillance	2/6	2/3	4/9
15	Complications/infection/nonunion follow-up	1/6	1/3	2/9
Total	All references	41/90	30/45	71/135

The 45 references generated by ChatGPT-5.3 corresponded to only 36 unique sources. A single article, the evidence-based review by Halawi and Morwood [6], was reproduced under 8 of the 15 topic categories (53.3%) and therefore accounted for 8 of the 45 references (17.8%). Two additional sources were each generated twice under different topic categories. Because the repeated review article received a score of 2 of 2 for bibliographic accuracy and 1 of 1 for content relevance on every occurrence, it accounted for 8 of the 11 references (72.7%) classified as fully bibliographically accurate. Across the entire dataset, only four unique sources achieved full bibliographic accuracy.

In the sensitivity analysis restricted to unique sources, the bibliographic accuracy rate decreased from 41/90 (45.6%) to 27/72 (37.5%), the content relevance rate from 30/45 (66.7%) to 23/36 (63.9%), and the overall performance score from 71/135 (52.6%) to 50/108 (46.3%). The comparison of the primary and sensitivity analyses is presented in Table 3.

**Table 3:** Comparison of the primary analysis and the sensitivity analysis restricted to unique sources. In the primary analysis, each generated reference was treated as a separate observation. In the sensitivity analysis, sources reproduced under more than one topic category contributed a single observation.

Measure	All generated references (*n* = 45)	Unique sources (*n* = 36)
Bibliographic accuracy	41/90 (45.6%)	27/72 (37.5%)
Content relevance	30/45 (66.7%)	23/36 (63.9%)
Overall performance score	71/135 (52.6%)	50/108 (46.3%)
Fully accurate references	11 (24.4%)	4 (11.1%)

## Discussion

In this study, we evaluated the bibliographic accuracy and content relevance of references that ChatGPT-5.3 presented as PubMed-indexed sources for open fracture management in orthopedic trauma. The main finding was that, although ChatGPT-5.3 organized management into commonly recognized domains of open fracture care that were broadly consistent with current principles, the references supporting these domains had limited bibliographic reliability. In this representative output, only 11 of 45 references (24.4%) were fully consistent with PubMed records; 19 (42.2%) existed but contained clear bibliographic errors, and 15 (33.3%) could not be verified as valid PubMed-indexed articles.

This indicates that ChatGPT-5.3’s ability to organize clinical information is not matched by an equivalent ability to support it with reliable academic sources. The model presented the main components of open fracture management coherently, including initial trauma assessment, limb examination, early antibiotic therapy, tetanus prophylaxis, irrigation and debridement, fracture stabilization, soft tissue coverage, vascular injury management, and complication follow-up. During reference generation, however, it produced citations that appeared plausible but contained incorrect PMIDs, erroneous journal or publication-year information, incomplete bibliographic details, or sources that could not be verified.

Another notable finding was that all bibliographically verifiable references were relevant to their assigned topics. This suggests that the model’s principal weakness was not the selection of clinically irrelevant literature but its inability to cite an otherwise appropriate body of work with the accuracy required for academic use. This distinction is important because it indicates that AI-generated outputs should be evaluated separately for clinical content and source reliability.

Previous studies have shown that ChatGPT and other large language models have substantial reliability problems when generating references for medical content. In the context of orthopedic trauma, Ghanem et al. evaluated 30 references generated by ChatGPT-4 for the ABCDE trauma approach and reported that only 43.3% were fully accurate, whereas 56.7% were inaccurate or nonexistent [8]. Bhattacharyya et al. evaluated 115 references from 30 short medical articles generated by ChatGPT-3.5 and found that 47% were fabricated, 46% were authentic but inaccurate, and only 7% were both authentic and accurate. In the same study, PMID errors were the most common error type and were observed in 93% of the generated papers [9]. Gravel et al. evaluated 59 references provided by ChatGPT for 20 medical questions and reported that 41 (69%) were fabricated. They emphasized that most fabricated references included realistic author names, plausible titles, and credible journal formats [10].

Applying the composite metric used by Ghanem et al., in which the total score is expressed as a proportion of the maximum attainable score, the overall bibliographic accuracy of ChatGPT-5.3 in the present study was 45.6%, compared with an average reference accuracy score of 66.7% reported for ChatGPT-4 in trauma protocol references [8]. This difference should not be interpreted as evidence of model regression because the two studies addressed different clinical topics, sampled different numbers of subdomains, used different reviewers, and differed in model configuration. These figures are therefore best interpreted as broadly consistent with the wide range of citation reliability reported across the AI literature, rather than as a direct, like-for-like comparison between model versions. However, the findings indicate that bibliographic reliability has not improved to a degree that would permit unverified use.

Similar problems have also been reported in other medical fields. In radiology, Wagner and Ertl-Wagner reported that 67% of ChatGPT-3 responses to daily clinical radiology questions were correct; however, only 36.2% of the 343 generated references could be identified through a literature search, while 63.8% were classified as unverifiable references. Among the 124 identifiable references, only 47 (37.9%) were reported to provide sufficient background information to correctly support the corresponding questions [11]. In the context of systematic reviews, Chelli et al. evaluated references generated by GPT-3.5, GPT-4, and Bard and reported hallucination rates of 39.6% for GPT-3.5, 28.6% for GPT-4, and 91.4% for Bard. They therefore emphasized that large language models should not be used as the primary or sole tools for systematic reviews or literature searches [12].

Studies aiming to systematically measure reference hallucination further support the importance of this issue. Aljamaan et al. developed the Reference Hallucination Score for medical AI chatbots and stated that reference verification should include separate assessment of components such as title, authors, journal, DOI, publication date, link, and topic relevance [13]. Alkaissi and McFarlane also showed that, in the context of scientific writing, ChatGPT could generate nonexistent article titles and PMID numbers belonging to different articles, highlighting AI hallucination as an important risk for academic source use [14].

Consistent with this literature, we also found that a substantial proportion of the references presented by ChatGPT-5.3 as PubMed-indexed sources were bibliographically inaccurate or unverifiable. However, the main feature distinguishing this work from previous research is that it evaluated ChatGPT-generated references in orthopedics in terms of both bibliographic accuracy and content relevance. This two-step assessment demonstrates not only whether a reference exists but also whether a bibliographically verifiable reference actually supports the corresponding open fracture management topic. As noted above, the limitation lay in citation accuracy rather than source selection.

Reference redundancy and citation concentration

A pattern not previously described in the orthopedic literature was the marked redundancy of the generated references. Rather than retrieving a distinct body of evidence for each of the 15 topic categories, ChatGPT-5.3 reproduced the same evidence-based review under more than half of them. This redundancy was not incidental to the accuracy estimates: the repeated source was one of only four articles the model reproduced with complete bibliographic accuracy, and it therefore accounted for the majority of the references classified as fully accurate. Reporting accuracy per generated reference consequently may overstate model performance because a single correctly recalled source is counted repeatedly across topics.

Previous work provides a partial framework for interpreting this behavior. Algaba et al. showed that reference lists generated from parametric knowledge alone, without search or retrieval augmentation, closely resemble human citation patterns but display a heightened bias toward highly cited work, a bias that persisted after adjustment for publication year, title length, publication venue, and number of authors. The same characteristics were observed in the nonexistent references generated by the models [15]. In the present study, the web search function was enabled; however, the recorded output did not indicate whether retrieval was actually invoked for this response. Therefore, neither the redundancy nor the fabrication observed here can be attributed with confidence to parametric recall alone. Pastucha et al., examining 1,947 references across six chatbot versions in otorhinolaryngology, reported that versions with web search enabled outperformed those relying on parametric knowledge alone, but that even the best-performing configuration remained below 60% accuracy [16]. The overall bibliographic accuracy observed in the present study falls within that range, indicating that access to live search does not by itself resolve the problem.

Complete bibliographic accuracy was achieved by only four unique sources, and it was not simply a function of how well known a source was. Several landmark articles that define this field were recalled with recognizable titles but erroneous bibliographic identifiers, including the trial of wound irrigation in open fractures, the original description of compartmental syndromes, the early microsurgical reconstruction series, and the fix-and-flap series. One widely cited review on bone and joint infection was cited with an identifier belonging to an unrelated article in a different field. One hypothesis consistent with this pattern is that the model retains the existence of prominent articles while reproducing their bibliographic identifiers unreliably; however, this cannot be established from a single output. Complete accuracy remained uncommon irrespective of a source’s prominence. Redundancy and inaccuracy thus represent two distinct failure modes: the model reuses a small number of sources it can reproduce correctly while generating identifiers it cannot substantiate for the remainder.

Descriptively, the eight topic categories in which the model reused the same review article showed lower bibliographic accuracy among their remaining references than the seven categories in which it did not (9 of 32 versus 16 of 42). Consistent with the descriptive approach adopted throughout, this comparison was not tested statistically and was based on a small number of observations; it should therefore be regarded as exploratory. Nevertheless, it raises the possibility that citation reuse functions as a fallback in topic areas where the model’s retrievable bibliographic knowledge is weakest, rather than as a deliberate selection of a broadly applicable source.

The prompt required three citations for every topic category, which imposed a fixed output quota irrespective of how much reliable literature could be retrieved for a given subdomain. This design ensured a comparable number of citations per topic category, but it may also have contributed to both fabrication and reuse: when fewer than three retrievable sources were available for a topic, the quota could be satisfied only by repeating a source the model could reproduce accurately or by generating an identifier it could not substantiate.

These observations have two practical implications for authors. First, published accuracy figures for AI-generated references should be interpreted in light of the redundancy of the underlying output, since per-reference rates may be inflated by repeated recall of a small number of sources. Second, the topics for which an author is most likely to require a literature search, namely narrower or less established clinical questions, may be precisely those for which the model is least reliable.

Open fracture management is a multistep and time-sensitive treatment process involving early antibiotic administration, tetanus prophylaxis, surgical irrigation and debridement, stabilization, soft tissue coverage, and complication surveillance [6]. In this context, a well-organized AI-generated response is not sufficient by itself; the sources supporting that response must also be verifiable. Failure to independently verify references may allow inaccurate citations to be incorporated into manuscripts, presentations, or educational materials. ChatGPT outputs, particularly in academic and clinical texts requiring supporting references, should therefore be used only alongside critical literature verification.

This distinction is clinically and academically important because a bibliographically inaccurate reference cannot be considered reliable, even when it appears to belong to a content-relevant area of the literature.

The high inter-rater agreement observed during the evaluation process supports the applicability and reproducibility of the scoring system. Nevertheless, content relevance assessment inherently involves a degree of interpretation. Content relevance was therefore evaluated only as a binary measure of whether the reference supported its assigned topic; the level of evidence and methodological quality of the articles were not analyzed separately.

This study has several limitations. First, the evaluation was limited to ChatGPT-5.3 and a single clinical topic: open fracture management in orthopedic trauma. The findings therefore cannot be generalized directly to other AI models, other versions of ChatGPT, or other orthopedic topics. Second, the queries were performed in March 2026 using two specific prompts. Because responses generated by large language models may vary according to model updates, session-related factors, and prompt structure, the same queries may produce different reference outputs at different time points. Third, because the model could reproduce the same source under more than one topic category, the 45 generated references were not fully independent observations. The primary analysis treated each reference as a separate unit, reflecting the output as it would be received by a user, and a sensitivity analysis restricted to unique sources was performed to address this dependency. This dependency also affects the inter-rater agreement estimates, which should therefore be interpreted as applying to the evaluation procedure rather than to a set of independent references. Fourth, the prompt required a fixed number of citations for every topic category. An open-ended prompt, or one permitting the model to indicate that no further sources could be retrieved, might yield different fabrication and redundancy rates.

Fifth, we evaluated the bibliographic accuracy and content relevance of the references but did not separately analyze the level of evidence, methodological quality, or weight of the cited articles in relation to clinical recommendations. Finally, content relevance was assessed using a binary scoring system. Although this approach was practical and applicable, it did not provide a detailed grading of the extent or strength with which each reference supported its assigned topic.

These findings suggest that large language models may be useful for organizing information and generating educational frameworks in orthopedic trauma; however, their citation-generating capacity still requires independent verification. Therefore, ChatGPT outputs, particularly in academic texts requiring PubMed-indexed references, should not be used as a substitute for a verified literature search but rather as a supportive starting point.

## Conclusions

In this single sampled output, although ChatGPT-5.3 organized open fracture management into commonly recognized clinical domains, the references it presented as PubMed-indexed sources to support this response showed substantial bibliographic reliability problems. All 30 bibliographically verifiable references were content-relevant to their corresponding topics. However, because a substantial proportion of the references contained inaccurate or unverifiable bibliographic information, ChatGPT-generated sources should not be relied upon for academic or clinical purposes without independent verification.

## Appendices

Appendix A

**Table 4:** Prompts submitted to ChatGPT-5.3

Query	Prompt text
First query	What is the management approach for open fractures in orthopedic trauma? Please describe the key steps systematically.
Follow-up query	Please provide PubMed-indexed references to support each of the steps mentioned above. For every topic give three citations.

Appendix B 

**Table 5:** Open fracture management framework generated by ChatGPT-5.3

No	Management domain	Response generated by ChatGPT-5.3
1	Initial Trauma Assessment / ATLS-DCO	The initial management of an open fracture should follow general trauma principles. The patient should be treated first, and the fracture second. Airway with cervical spine protection, breathing, circulation with hemorrhage control, disability or neurologic status, and exposure should be assessed according to ATLS principles. In polytrauma or physiologically unstable patients, damage-control orthopedic principles should be considered. Life-threatening injuries, hemorrhage, shock, and systemic instability should be addressed before definitive fracture reconstruction.
2	Limb Assessment / Neurovascular Examination	After initial stabilization, the injured limb should be systematically evaluated. The wound size, location, degree of contamination, bone exposure, soft tissue loss, and associated tendon or muscle injury should be documented. A careful neurovascular examination is essential. Distal pulses, capillary refill, skin temperature, motor function, and sensory function should be assessed and recorded before intervention. Clinical signs of compartment syndrome should also be actively sought.
3	Sterile Dressing / Temporary Immobilization	The open wound should be covered promptly with a sterile dressing. Gross contaminants may be gently removed or lightly irrigated with sterile saline, but repeated probing or aggressive emergency department washout should be avoided. A sterile saline-soaked dressing and sterile bandage should be applied, and the limb should be immobilized with an appropriate splint. Bedside wound closure should not be attempted, especially when contamination or soft tissue compromise is present.
4	Early Intravenous Antibiotics	Intravenous antibiotics should be administered as early as possible, ideally within the first hour after presentation. Early antibiotic administration is one of the most important modifiable factors for reducing infection risk in open fractures. Antibiotic treatment should not be delayed for imaging, operative planning, or definitive classification. The initial regimen should be started promptly and later adjusted according to injury severity, contamination type, institutional protocol, and culture results when relevant.
5	Antibiotic Selection by Gustilo Severity	Antibiotic selection is usually guided by the severity of the open fracture and the contamination pattern. Gustilo type I and II injuries are commonly treated with a first-generation cephalosporin such as cefazolin. For Gustilo type III injuries, additional gram-negative coverage may be required according to local protocols. Farm, fecal, or soil contamination may require anaerobic coverage. Water-related injuries require tailored antimicrobial coverage based on the exposure environment.
6	Tetanus Prophylaxis	Tetanus immunization status should be assessed in every patient with an open fracture. A tetanus booster should be given when indicated. In patients with incomplete or unknown vaccination status and a contaminated or dirty wound, tetanus immune globulin should be considered in addition to vaccination.
7	Imaging	Radiographic evaluation should include anteroposterior and lateral views of the injured segment, ideally including the joint above and below the fracture. Imaging helps define fracture morphology, displacement, bone loss, and associated joint involvement. Computed tomography may be useful when there is suspected intra-articular extension, complex periarticular anatomy, or a need for detailed preoperative planning. However, imaging should not delay urgent antibiotic administration or necessary operative treatment.
8	Gustilo-Anderson Classification	The Gustilo-Anderson classification remains the most commonly used system for grading open fractures. Type I injuries involve small, relatively clean wounds; type II injuries involve larger wounds with moderate soft tissue damage; and type III injuries involve severe soft tissue injury, contamination, periosteal stripping, or vascular compromise. Type III injuries are further divided into IIIA, IIIB, and IIIC. Definitive classification is most accurate after operative exploration and debridement, because the true extent of soft tissue injury may not be apparent during the initial emergency department examination.
9	Irrigation and Surgical Debridement	Operative irrigation and debridement are central components of open fracture management. The goals are to remove devitalized tissue, foreign material, and contaminants; reduce bacterial burden; and reassess the fracture and soft tissue envelope. Debridement should be thorough and systematic. The wound may need to be extended for adequate exposure. Nonviable skin, fascia, muscle, and bone should be excised. Repeat debridement may be required when tissue viability remains uncertain. The traditional “6-hour rule” is no longer considered absolute; urgency should be determined by contamination, vascular status, compartment syndrome, and overall injury severity.
10	Fracture Stabilization	Fracture stabilization is important for pain control, soft tissue recovery, infection control, and later functional rehabilitation. The method of stabilization should be selected according to fracture pattern, soft tissue condition, contamination, patient physiology, and planned reconstruction. Options include temporary splinting, external fixation, internal fixation with plates or intramedullary nails, and staged fixation in damage-control settings. In unstable polytrauma patients, temporary external fixation may be preferred before definitive fixation.
11	Soft Tissue Coverage / Flaps	Soft tissue management is an integral part of open fracture treatment. The wound should not be closed prematurely if contamination, devitalized tissue, or soft tissue uncertainty remains. Coverage options include delayed primary closure, split-thickness skin grafting, local flap coverage, and free tissue transfer. For severe open fractures, especially Gustilo type IIIB injuries, early coordinated orthoplastic management and timely flap coverage are important for reducing complications and improving outcomes.
12	Negative Pressure Wound Therapy	Negative pressure wound therapy may be used as an adjunct in selected open fractures, particularly when immediate definitive closure or flap coverage is not possible. It can help manage the wound environment temporarily between debridement and definitive soft tissue coverage. However, negative pressure wound therapy should not be considered a substitute for adequate debridement, stable fixation, or timely soft tissue reconstruction. Its use should be integrated into a staged wound management plan.
13	Vascular Injury / Ischemic Limb	Open fractures associated with vascular injury require urgent recognition and management. Absent pulses, expanding hematoma, active arterial bleeding, distal ischemia, or neurologic compromise should prompt immediate vascular assessment. Initial reduction and splinting may restore perfusion in some cases. If ischemia persists, urgent vascular surgery consultation, vascular imaging when appropriate, temporary shunting, and revascularization should be considered. Warm ischemia time is critical, and limb salvage decisions should be made in the context of the overall injury pattern and patient condition.
14	Compartment Syndrome Surveillance	Patients with open fractures remain at risk for acute compartment syndrome, even when the wound is open. Pain out of proportion, pain with passive stretch, tense compartments, neurologic symptoms, and deteriorating perfusion should raise concern. Serial clinical examinations are essential. When the diagnosis is uncertain, compartment pressure measurement may be used. If compartment syndrome is suspected, urgent fasciotomy should not be delayed.
15	Complications / Infection / Nonunion Follow-up	Open fractures require close follow-up for early and late complications. Early complications include infection, soft tissue necrosis, compartment syndrome, vascular compromise, and thromboembolic events. Late complications include delayed union, nonunion, malunion, chronic osteomyelitis, stiffness, and post-traumatic arthritis. Follow-up should assess wound healing, infection signs, fixation stability, bone union, limb alignment, joint motion, and functional recovery.

Appendix C 

**Table 6:** References generated by ChatGPT-5.3 (n = 45)

Ref.	Topic category	Reference exactly as generated by ChatGPT-5.3
1.1	Initial Trauma Assessment (ATLS / Damage Control)	American College of Surgeons Committee on Trauma. Advanced Trauma Life Support (ATLS) principles in orthopedic trauma care. Widely accepted trauma framework. PMID-indexed trauma literature supports ABC prioritization.
1.2	Initial Trauma Assessment (ATLS / Damage Control)	Giannoudis PV, et al. Damage control orthopaedics in unstable polytrauma patients. J Bone Joint Surg Br. 2003. PMID: 12678358
1.3	Initial Trauma Assessment (ATLS / Damage Control)	Pape HC, et al. Timing of fracture fixation in multitrauma patients. J Am Acad Orthop Surg. 2005. PMID: 15712904
2.1	Limb Assessment / Neurovascular Examination	Halawi MJ, Morwood MP. Acute Management of Open Fractures: An Evidence-Based Review. Orthopedics. 2015. PMID: 26558667
2.2	Limb Assessment / Neurovascular Examination	Rockwood and Green's Fractures in Adults (clinical principles reflected in PubMed trauma reviews)
2.3	Limb Assessment / Neurovascular Examination	Brinker MR, O'Connor DP. Assessment of limb-threatening extremity trauma. Instr Course Lect. PMID: 19385542
3.1	Sterile Dressing / Temporary Immobilization	Halawi MJ, Morwood MP. Acute Management of Open Fractures: An Evidence-Based Review. Orthopedics. 2015;38(11):e1025-e1033. doi:10.3928/01477447-20151020-12. PMID: 26558667.
3.2	Sterile Dressing / Temporary Immobilization	Pollak AN, et al. Temporary stabilization methods in severe extremity trauma. J Am Acad Orthop Surg. PMID: 12828449
3.3	Sterile Dressing / Temporary Immobilization	Court-Brown CM, et al. Initial management principles of open fractures. Injury. PMID: 11311731
4.1	Early Intravenous Antibiotics	Patzakis MJ, Wilkins J. Factors influencing infection rate in open fracture wounds. Clin Orthop Relat Res. 1989. PMID: 2721073
4.2	Early Intravenous Antibiotics	Lack WD, et al. Type III open tibia fractures: immediate antibiotics reduce infection. J Orthop Trauma. 2015. PMID: 25581616
4.3	Early Intravenous Antibiotics	Halawi MJ, Morwood MP. Acute Management of Open Fractures: An Evidence-Based Review. Orthopedics. 2015;38(11):e1025-e1033. doi:10.3928/01477447-20151020-12. PMID: 26558667.
5.1	Antibiotic Selection by Gustilo Severity	Hoff WS, et al. Practice management guidelines for prophylactic antibiotic use in open fractures. J Trauma. PMID: 15094570
5.2	Antibiotic Selection by Gustilo Severity	Hauser CJ, et al. Antibiotic management of open fractures. Surg Infect. PMID: 19673668
5.3	Antibiotic Selection by Gustilo Severity	Halawi MJ, Morwood MP. Acute Management of Open Fractures: An Evidence-Based Review. Orthopedics. 2015;38(11):e1025-e1033. doi:10.3928/01477447-20151020-12. PMID: 26558667.
6.1	Tetanus Prophylaxis	CDC / tetanus wound management recommendations (PubMed-indexed review articles summarize these protocols)
6.2	Tetanus Prophylaxis	Edlich RF, et al. Management and prevention of tetanus. J Long Term Eff Med Implants. PMID: 11883546
6.3	Tetanus Prophylaxis	Halawi MJ, Morwood MP. Acute Management of Open Fractures: An Evidence-Based Review. Orthopedics. 2015;38(11):e1025-e1033. doi:10.3928/01477447-20151020-12. PMID: 26558667.
7.1	Imaging (X-ray / CT / CTA)	Halawi MJ, Morwood MP. Acute Management of Open Fractures: An Evidence-Based Review. Orthopedics. 2015;38(11):e1025-e1033. doi:10.3928/01477447-20151020-12. PMID: 26558667.
7.2	Imaging (X-ray / CT / CTA)	Tornetta P III, et al. CT role in periarticular fracture planning. J Orthop Trauma. PMID: 15213497
7.3	Imaging (X-ray / CT / CTA)	Inaba K, et al. CTA for extremity vascular trauma. J Trauma. PMID: 18376169
8.1	Gustilo-Anderson Classification	Gustilo RB, Anderson JT. Prevention of infection in treatment of one thousand and twenty-five open fractures. J Bone Joint Surg Am. 1976. PMID: 773941
8.2	Gustilo-Anderson Classification	Gustilo RB, et al. Problems in management of type III open fractures. J Trauma. 1984. PMID: 6708156
8.3	Gustilo-Anderson Classification	Kim PH, Leopold SS. Gustilo-Anderson classification review. Clin Orthop Relat Res. PMID: 22207507
9.1	Irrigation and Surgical Debridement	FLOW Investigators. A trial of wound irrigation in initial management of open fractures. N Engl J Med. 2015. PMID: 26465983
9.2	Irrigation and Surgical Debridement	Anglen JO. Wound irrigation in musculoskeletal injury. J Am Acad Orthop Surg. PMID: 11281638
9.3	Irrigation and Surgical Debridement	Halawi MJ, Morwood MP. Acute Management of Open Fractures: An Evidence-Based Review. Orthopedics. 2015;38(11):e1025-e1033. doi:10.3928/01477447-20151020-12. PMID: 26558667.
10.1	Fracture Stabilization (External / Internal Fixation)	Bhandari M, et al. Reamed vs unreamed nailing in tibial fractures. J Bone Joint Surg Am. PMID: 16322610
10.2	Fracture Stabilization (External / Internal Fixation)	Giannoudis PV, et al. External fixation in damage-control orthopedics. PMID: 12678358
10.3	Fracture Stabilization (External / Internal Fixation)	Halawi MJ, Morwood MP. Acute Management of Open Fractures: An Evidence-Based Review. Orthopedics. 2015;38(11):e1025-e1033. doi:10.3928/01477447-20151020-12. PMID: 26558667.
11.1	Soft Tissue Coverage / Flaps	Godina M. Early microsurgical reconstruction of complex trauma of the extremities. Plast Reconstr Surg. PMID: 3889367
11.2	Soft Tissue Coverage / Flaps	Gopal S, et al. Fix and flap concept in severe open tibial fractures. J Bone Joint Surg Br. PMID: 11132261
11.3	Soft Tissue Coverage / Flaps	Pollak AN, et al. Delay in flap coverage increases complications. J Bone Joint Surg Am. PMID: 15118029
12.1	Negative Pressure Wound Therapy (Adjunct)	Costa ML, et al. WOLLF trial: NPWT vs standard dressings in severe open fractures. BMJ. PMID: 29896626
12.2	Negative Pressure Wound Therapy (Adjunct)	Stannard JP, et al. NPWT after severe open fractures. J Orthop Trauma. PMID: 19318864
12.3	Negative Pressure Wound Therapy (Adjunct)	Blum ML, et al. NPWT systematic review in open tibia fractures. Injury. PMID: 22840312
13.1	Vascular Injury / Ischemic Limb	Fox N, et al. Evaluation and management of penetrating/extremity vascular trauma. J Trauma Acute Care Surg. PMID: 29319660
13.2	Vascular Injury / Ischemic Limb	Helfet DL, et al. Limb salvage after vascular injury with fractures. Clin Orthop Relat Res. PMID: 7634671
13.3	Vascular Injury / Ischemic Limb	Johansen K, et al. Mangled extremity severity score. J Trauma. PMID: 2342140
14.1	Compartment Syndrome Surveillance	Olson SA, Glasgow RR. Acute compartment syndrome in lower extremity trauma. J Am Acad Orthop Surg. PMID: 16467184
14.2	Compartment Syndrome Surveillance	McQueen MM, Duckworth AD. Diagnosis and management of compartment syndrome. Bone Joint J. PMID: 24395314
14.3	Compartment Syndrome Surveillance	Matsen FA III. Compartmental syndromes. N Engl J Med. PMID: 7466320
15.1	Complications / Infection / Nonunion Follow-up	Metsemakers WJ, et al. Fracture-related infection definition and management. Injury. PMID: 28843446
15.2	Complications / Infection / Nonunion Follow-up	Zimmerli W. Clinical practice: bone and joint infections. N Engl J Med. PMID: 16554528
15.3	Complications / Infection / Nonunion Follow-up	Court-Brown CM, et al. Open fracture outcomes and complications. Injury. PMID: 11311731

Appendix D

**Table 7:** Reference-level bibliographic verification and content-relevance assessment (n = 45)

No	Reference as generated by ChatGPT-5.3	Bibliographic accuracy	Content relevance
1.1	American College of Surgeons Committee on Trauma. ATLS principles in orthopedic trauma care	0/2. This was not written in the form of a real article reference. It does not include an author, title, journal, year, volume/issue/pages, or PMID. The ACS ATLS is real as an educational/guideline system; however, this statement is not a PubMed-indexed article reference. There are articles related to ATLS in PubMed; for example, the publication titled “Advanced trauma life support (ATLS®): the ninth edition” is associated with the ACS Committee on Trauma and appears with PMID 23609291. However, this is not the provided reference itself.	0/1. Because the provided source is not defined as a PubMed article, an abstract review cannot be performed. Although the ATLS approach is conceptually appropriate for the initial assessment of an open fracture, this item is not usable as a reference.
1.2	Giannoudis PV, et al. Damage control orthopaedics in unstable polytrauma patients. J Bone Joint Surg Br. 2003. PMID: 12678358	0/2. The provided PMID is completely incorrect. PMID 12678358 does not refer to damage-control orthopedics, but to the following article: Holzer G, Krepler P, Koschat MA, Grampp S, Dominkus M, Kotz R. Bone mineral density in long-term survivors of highly malignant osteosarcoma. J Bone Joint Surg Br. 2003;85(2):231-237. PMID: 12678358. The PubMed page clearly shows the title, authors, and PMID in this way. A Giannoudis record more relevant to damage-control orthopedics is: Giannoudis PV, Pape HC. Damage control orthopaedics in unstable pelvic ring injuries. Injury. 2004;35(7):671-677. PMID: 15203307. Search results indicate that this article addresses the damage-control orthopedics approach in unstable/in extremis multitrauma patients.	0/1. Content relevance could not be assessed because the reference was not bibliographically verifiable.
1.3	Pape HC, et al. Timing of fracture fixation in multitrauma patients. J Am Acad Orthop Surg. 2005. PMID: 15712904	1/2. The provided information appears to be incorrect. The reliable PubMed match for this title is: Pape HC, Tornetta P 3rd, Tarkin I, Tzioupis C, Sabeson V, Olson SA. Timing of fracture fixation in multitrauma patients: the role of early total care and damage control surgery. J Am Acad Orthop Surg. 2009;17(9):541-549. PMID: 19726738. The provided record states the year as 2005 and the PMID as 15712904; the correct record appears to be 2009 with PMID 19726738.	1/1. The article states that the timing of fracture stabilization in patients with multitrauma is controversial; that early definitive surgery has advantages in most patients; but that in selected patients with severe multisystem injury, early temporary stabilization with an external fixator followed by definitive fixation may provide a survival advantage.
2.1	Halawi MJ, Morwood MP. Acute Management of Open Fractures: An Evidence-Based Review. Orthopedics. 2015. PMID: 26558667	2/2. Consistent with the PubMed record: Halawi MJ, Morwood MP. Acute Management of Open Fractures: An Evidence-Based Review. Orthopedics. 2015;38(11):e1025-e1033. PMID: 26558667.	1/1. The article states that open fracture management should begin with systematic assessment and management after stabilization of life-threatening conditions. It also addresses topics such as irrigation and debridement, antibiotics, soft tissue management, and early closure. This article is directly appropriate for the acute management of open fractures. It may support several headings, including limb assessment, antibiotics, debridement, stabilization, and soft tissue management.
2.2	Rockwood and Green’s Fractures in Adults	0/2. This is a book source; it is not a PubMed-indexed article. ChatGPT cannot access and read medical textbook sources.	0/1. Content relevance could not be assessed because the reference was not bibliographically verifiable.
2.3	Brinker MR, O'Connor DP. Assessment of limb-threatening extremity trauma. Instr Course Lect. PMID: 19385542	0/2. The provided reference could not be matched to any PubMed record. PMID 19385542 does not belong to Brinker and O'Connor or to an article on the assessment of limb-threatening extremity trauma; it corresponds to an unrelated review published in Instructional Course Lectures in 2009. No PubMed record matching the provided author, title, and journal combination was identified.	0/1. Content relevance could not be assessed because the reference was not bibliographically verifiable.
3.1	Halawi MJ, Morwood MP. Acute Management of Open Fractures: An Evidence-Based Review. Orthopedics. 2015;38(11):e1025-e1033. doi:10.3928/01477447-20151020-12. PMID: 26558667.	2/2. This reference is correct. PubMed record: Halawi MJ, Morwood MP. Acute Management of Open Fractures: An Evidence-Based Review. Orthopedics. 2015;38(11):e1025-e1033. doi:10.3928/01477447-20151020-12. PMID: 26558667.	1/1. The article discusses emergency management of open fractures through core headings such as patient stabilization, systematic assessment, antibiotics, irrigation and debridement, soft tissue management, and early closure. Therefore, it is suitable as a general reference to support initial management steps such as sterile dressing/splinting. Directly related to the acute management of open fractures.
3.2	Pollak AN, et al. Temporary stabilization methods in severe extremity trauma. J Am Acad Orthop Surg. PMID: 12828449	0/2. The provided reference could not be matched to any PubMed record. PMID 12828449 does not belong to Pollak et al. or to an article on temporary stabilization methods in severe extremity trauma; it corresponds to a review on quadriceps tendon rupture published in the same journal. No PubMed record matching the provided author, title, and journal combination was identified.	0/1. Content relevance could not be assessed because the reference was not bibliographically verifiable.
3.3	Court-Brown CM, et al. Initial management principles of open fractures. Injury. PMID: 11311731	0/2. The provided reference could not be matched to any PubMed record. As also noted for reference 15.3, PMID 11311731 is not related to Court-Brown or to open fracture management; it belongs to an unrelated pharmacology article. The same identifier was generated by the model under two different topic categories. No PubMed record matching the provided author, title, and journal combination was identified.	0/1. Content relevance could not be assessed because the reference was not bibliographically verifiable.
4.1	Patzakis MJ, Wilkins J. Factors influencing infection rate in open fracture wounds. Clin Orthop Relat Res. 1989. PMID: 2721073	2/2. The reference is correct. PubMed/Europe PMC record: Patzakis MJ, Wilkins J. Factors influencing infection rate in open fracture wounds. Clin Orthop Relat Res. 1989;(243):36-40. PMID: 2721073.	1/1. The article evaluated infections that developed in 1104 open fracture wounds and stated that the most important factor reducing infection rates was early antibiotic administration. It also states that surgical debridement was performed in all open fractures. A directly appropriate and classic reference supporting early intravenous antibiotic administration.
4.2	Lack WD, et al. Type III open tibia fractures: immediate antibiotics reduce infection. J Orthop Trauma. 2015. PMID: 25581616	1/2. The article cited in the reference exists, but the PMID is incorrect. The provided PMID 25581616 belongs to the following article: Yao X, et al. Oral Delivery of Bovine Lactoferrin Using Pectin- and Chitosan-Modified Liposomes and Solid Lipid Particles: Improvement of Stability of Lactoferrin. Chem Biol Drug Des. 2015. PMID: 25581616. This is not related to open fractures. The correct PubMed record for this reference is as follows: Lack WD, Karunakar MA, Angerame MR, Seymour RB, Sims S, Kellam JF, Bosse MJ. Type III open tibia fractures: immediate antibiotic prophylaxis minimizes infection. J Orthop Trauma. 2015;29(1):1-6. PMID: 25526095.	1/1. The article examined the relationship between antibiotic timing and deep infection in type III open tibia fractures. This directly supports the “early antibiotics” heading. The article directly investigates whether early antibiotic prophylaxis reduces infection in open tibia fractures. The article directly investigates whether early antibiotic prophylaxis reduces infection in open tibia fractures.
4.3	Halawi MJ, Morwood MP. Acute Management of Open Fractures: An Evidence-Based Review. Orthopedics. 2015;38(11):e1025-e1033. doi:10.3928/01477447-20151020-12. PMID: 26558667.	2/2. The reference is consistent with PubMed: Halawi MJ, Morwood MP. Acute Management of Open Fractures: An Evidence-Based Review. Orthopedics. 2015;38(11):e1025-e1033. doi:10.3928/01477447-20151020-12. PMID: 26558667.	1/1. The article discusses acute management steps in open fracture management, including antibiotics, irrigation and debridement, soft tissue management, early closure, antibiotic-impregnated implants/devices, and suspicion of compartment syndrome. Directly appropriate for the acute management of open fractures. It supports multiple management steps, including the early antibiotic heading.
5.1	Hoff WS, et al. Practice management guidelines for prophylactic antibiotic use in open fractures. J Trauma. PMID: 15094570	1/2. The topic of the reference is correct, but the provided PMID is incorrect. The provided PMID 15094570 does not belong to this article; no open fracture antibiotic guideline matched this PMID in PubMed search. The correct record is as follows: Hoff WS, Bonadies JA, Cachecho R, Dorlac WC; East Practice Management Guidelines Work Group. East Practice Management Guidelines Work Group: update to practice management guidelines for prophylactic antibiotic use in open fractures. J Trauma. 2011;70(3):751-754. PMID: 21610369.	1/1. The article updates the practice management guideline for prophylactic antibiotic use in open fractures; in this clinical context, it directly addresses antibiotic selection, timing of administration, and prophylaxis strategy. It is directly appropriate for antibiotic prophylaxis in open fractures.
5.2	Hauser CJ, et al. Antibiotic management of open fractures. Surg Infect. PMID: 19673668	1/2. The topic of the provided reference is close to correct, but the PMID is incorrect and the title is incomplete/incorrectly given. The provided PMID 19673668 belongs to the following article: Globas C, et al. Role of walking-exercise therapy after stroke. Expert Rev Cardiovasc Ther. 2009. PMID: 19673668. This is not related to open fractures. The correct record should be: Hauser CJ, Adams CA Jr, Eachempati SR; Surgical Infection Society. Prophylactic Antibiotic Use in Open Fractures: An Evidence-Based Guideline. Surg Infect (Larchmt). 2006;7(4):379-405. PMID: 16978082.	1/1. The article systematically evaluates the effects of prophylactic antibiotic administration for preventing infectious complications in open fractures. The study is an evidence-based guideline on antibiotic prophylaxis in open fractures. It is appropriate for the antibiotic selection/prophylaxis heading in open fractures. It is appropriate for the antibiotic selection/prophylaxis heading in open fractures.
5.3	Halawi MJ, Morwood MP. Acute Management of Open Fractures: An Evidence-Based Review. Orthopedics. 2015;38(11):e1025-e1033. doi:10.3928/01477447-20151020-12. PMID: 26558667.	2/2. Reference correct: Halawi MJ, Morwood MP. Acute Management of Open Fractures: An Evidence-Based Review. Orthopedics. 2015;38(11):e1025-e1033. doi:10.3928/01477447-20151020-12. PMID: 26558667.	1/1. The article evaluates the main components of acute management in open fractures; these include antibiotics and discussions related to prophylaxis for Gustilo type III open fractures. It is appropriate for antibiotic selection and general open fracture management.
6.1	CDC / tetanus wound management recommendations	0/2. This statement is not a PubMed-indexed article reference. CDC recommendations are clinically valuable, but what is provided here is not an article citation; it lacks author, title, journal, year, and PMID. Articles related to tetanus prophylaxis can be found in PubMed; for example, the StatPearls/NCBI record titled “Tetanus Prophylaxis” is indexed with PMID 32644434. However, this is not the provided reference itself.	0/1. Content relevance could not be assessed because the reference was not bibliographically verifiable.
6.2	Edlich RF, et al. Management and prevention of tetanus. J Long Term Eff Med Implants. PMID: 11883546	1/2. The title and author of the provided reference appear to be real; however, the PMID is incorrect. The correct record in PubMed is as follows: Edlich RF, Hill LG, Mahler CA, Cox MJ, Becker DG, Horowitz JH, Nichter LS, Martin ML, Lineaweaver WC. Management and prevention of tetanus. J Long Term Eff Med Implants. 2003;13(3):139-154. PMID: 14516181. The provided PMID 11883546 does not match this article.	1/1. The article addresses the clinical management of tetanus and immunization strategies for tetanus prevention; therefore, it supports headings such as tetanus prophylaxis after contaminated wounds, assessment of immunization status, and booster vaccination/immunoglobulin administration when indicated. It is appropriate for the tetanus prophylaxis/management heading. It is not specific to open fractures, but it supports the tetanus prophylaxis component of contaminated wound management in open fractures.
6.3	Halawi MJ, Morwood MP. Acute Management of Open Fractures: An Evidence-Based Review. Orthopedics. 2015;38(11):e1025-e1033. doi:10.3928/01477447-20151020-12. PMID: 26558667.	2/2. The reference is consistent with PubMed: Halawi MJ, Morwood MP. Acute Management of Open Fractures: An Evidence-Based Review. Orthopedics. 2015;38(11):e1025-e1033. doi:10.3928/01477447-20151020-12. PMID: 26558667.	1/1. The article states that open fracture management should begin systematically after life-threatening conditions have been stabilized; it discusses antibiotics, irrigation and debridement, soft tissue management, and other acute management headings. In addition, the article abstract/index information indicates that tetanus prophylaxis is also one of the practical management headings within acute open fracture management. It is directly related to open fracture management and is suitable to support the initial acute steps, including tetanus prophylaxis.
7.1	Halawi MJ, Morwood MP. Acute Management of Open Fractures: An Evidence-Based Review. Orthopedics. 2015;38(11):e1025-e1033. doi:10.3928/01477447-20151020-12. PMID: 26558667.	2/2. The same PubMed record is correct: Halawi MJ, Morwood MP. Acute Management of Open Fractures: An Evidence-Based Review. Orthopedics. 2015;38(11):e1025-e1033. doi:10.3928/01477447-20151020-12. PMID: 26558667.	1/1. The article comprehensively discusses the acute assessment and management of open fractures and supports basic steps such as initial assessment, antibiotic therapy, surgical debridement, stabilization, and soft tissue management. However, it is not a source that directly centers on detailed radiologic protocols such as AP/lateral radiographs, imaging of the joint above and below, or CT indications; therefore, under the imaging heading, it can only be used as a general supporting reference. Because it is related to the acute management of open fractures, it can generally support the imaging step. However, it would be more appropriate to support this heading with a more specific imaging article.
7.2	Tornetta P III, et al. CT role in periarticular fracture planning. J Orthop Trauma. PMID: 15213497	0/2. The provided reference does not match PubMed. PMID 15213497 does not belong to Tornetta or to an article on the role of CT in periarticular fracture planning. Reliable sources in which this PMID appears show that it belongs to the following article: Høiness P, Strømsøe K. Tricortical versus quadricortical syndesmosis fixation in ankle fractures: a prospective, randomized study comparing two methods of syndesmosis fixation. J Orthop Trauma. 2004;18(6):331-337. PMID: 15213497. Therefore, the provided title/author/PMID match is incorrect.	0/1. Content relevance could not be assessed because the reference was not bibliographically verifiable.
7.3	Inaba K, et al. CTA for extremity vascular trauma. J Trauma. PMID: 18376169	0/2. In its provided form, the reference does not match PubMed. I could not find an “Inaba / CTA / extremity vascular trauma” match for PMID 18376169. The clearly matching Inaba studies are as follows: 1. Inaba K, et al. Multi-slice CT Angiography for Arterial Evaluation in the Injured Lower Extremity. J Trauma. 2006. PMID: 16531846. 2. Inaba K, et al. Prospective evaluation of multidetector computed tomographic angiography for extremity vascular trauma. J Trauma. 2011. PMID: 21610388. Therefore, the provided author/topic may be close to correct, but the PMID is incorrect.	0/1. Content relevance could not be assessed because the reference was not bibliographically verifiable.
8.1	Gustilo RB, Anderson JT. Prevention of infection in treatment of one thousand and twenty-five open fractures. J Bone Joint Surg Am. 1976. PMID: 773941	2/2. The reference is consistent with PubMed. Correct title: Gustilo RB, Anderson JT. Prevention of infection in the treatment of one thousand and twenty-five open fractures of long bones: retrospective and prospective analyses. J Bone Joint Surg Am. 1976;58(4):453-458. PMID: 773941.	1/1. The article evaluates prevention of infection in 1025 open long-bone fractures through retrospective and prospective analyses and demonstrates the relationship between open fracture classification and infection risk. Therefore, it can be used as the foundational historical source for the Gustilo-Anderson classification.
8.2	Gustilo RB, et al. Problems in management of type III open fractures. J Trauma. 1984. PMID: 6708156	1/2. The provided reference is close to correct, but the PMID is incorrect. Correct PubMed record: Gustilo RB, Mendoza RM, Williams DN. Problems in the management of type III severe open fractures: a new classification of type III open fractures. J Trauma. 1984;24(8):742-746. PMID: 6471139. In addition, there is another Gustilo classification article from 1987 in PubMed: Gustilo RB, et al. Classification of type III severe open fractures relative to treatment and results. Orthopedics. 1987. PMID: 3324085.	1/1. The article emphasizes that the type III open fracture category is heterogeneous in terms of clinical severity and prognosis, and proposes a new classification of type III open fractures by dividing this broad group into more meaningful subgroups. Therefore, it is appropriate for supporting the IIIA, IIIB, and IIIC distinctions in the Gustilo-Anderson classification.
8.3	Kim PH, Leopold SS. Gustilo-Anderson classification review. Clin Orthop Relat Res. PMID: 22207507	1/2. The authors and topic area of the provided reference are correct, but the PMID is incorrect. Correct PubMed record: Kim PH, Leopold SS. In brief: Gustilo-Anderson classification. Clin Orthop Relat Res. 2012;470(11):3270-3274. PMID: 22569719.	1/1. The article presents the Gustilo-Anderson classification as a fundamental classification system that helps assess prognosis, guide treatment decisions, and establish a standardized communication language among surgeons in open fractures. Therefore, it is appropriate for the open fracture classification heading. It is appropriate for explaining the Gustilo-Anderson classification.
9.1	FLOW Investigators. A trial of wound irrigation in initial management of open fractures. N Engl J Med. 2015. PMID: 26465983	1/2. The topic and study of the provided reference are correct, but the PMID is incorrect. The correct PubMed record is as follows: FLOW Investigators; Bhandari M, et al. A Trial of Wound Irrigation in the Initial Management of Open Fracture Wounds. N Engl J Med. 2015;373(27):2629-2641. PMID: 26448371. The article appears with this PMID in PubMed search. The provided PMID 26465983 does not match this article.	1/1. The article addresses the irrigation and debridement step used to reduce contamination in open fracture wounds and compares the effects of different irrigation pressures and castile soap versus saline solution on clinical outcomes. Therefore, it is a source that directly supports the irrigation and debridement heading in open fractures.
9.2	Anglen JO. Wound irrigation in musculoskeletal injury. J Am Acad Orthop Surg. PMID: 11281638	1/2. The provided reference is very close to correct, but the PMID is incorrect. Correct PubMed record: Anglen JO. Wound irrigation in musculoskeletal injury. J Am Acad Orthop Surg. 2001;9(4):219-226. PMID: 11476531. The provided PMID 11281638 does not match this article.	1/1. The article states that wound irrigation is a fundamental component of open fracture care for removing debris and reducing bacterial contamination. It also states that there is insufficient evidence regarding irrigation fluid and method; therefore, most recommendations are based on animal and laboratory studies. It is appropriate for supporting irrigation principles in open fractures. It is appropriate for supporting irrigation principles in open fractures.
9.3	Halawi MJ, Morwood MP. Acute Management of Open Fractures: An Evidence-Based Review. Orthopedics. 2015;38(11):e1025-e1033. doi:10.3928/01477447-20151020-12. PMID: 26558667.	2/2. The reference was verified previously: Halawi MJ, Morwood MP. Acute Management of Open Fractures: An Evidence-Based Review. Orthopedics. 2015;38(11):e1025-e1033. doi:10.3928/01477447-20151020-12. PMID: 26558667.	1/1. The article addresses the basic steps of open fracture management, including antibiotics, irrigation and debridement, soft tissue management, and early closure. Therefore, it is suitable as a general management reference for the surgical debridement and irrigation heading. It is directly related to the acute management of open fractures. It is directly related to the acute management of open fractures. 10. Fracture Stabilization (External / Internal Fixation)
10.1	Bhandari M, et al. Reamed vs unreamed nailing in tibial fractures. J Bone Joint Surg Am. PMID: 16322610	1/2. The provided PMID is completely incorrect. PMID 16322610 does not belong to Bhandari’s tibial nailing study, but to the following article: Farrell CM, Springer BD, Haidukewych GJ, Morrey BF. Motor nerve palsy following primary total hip arthroplasty. J Bone Joint Surg Am. 2005;87(12):2619-2625. PMID: 16322610. The correct record for Bhandari’s reamed/unreamed tibial shaft fracture RCT is as follows: Bhandari M, et al. Randomized trial of reamed and unreamed intramedullary nailing of tibial shaft fractures. J Bone Joint Surg Am. 2008;90(12):2567-2578. PMID: 19047701. In addition, the PubMed record for the SPRINT study protocol/explanatory publication is PMID 18573205.	1/1. The correct Bhandari RCT article states that reamed and unreamed intramedullary nailing in tibial shaft fractures were compared in terms of reoperation and complication rates. Therefore, it is an appropriate reference for the surgical treatment of tibial shaft fractures.
10.2	Giannoudis PV, et al. External fixation in damage-control orthopedics. PMID: 12678358	0/2. The provided PMID is completely incorrect. PMID 12678358 does not refer to damage-control orthopedics, but to the following article: Holzer G, Krepler P, Koschat MA, Grampp S, Dominkus M, Kotz R. Bone mineral density in long-term survivors of highly malignant osteosarcoma. J Bone Joint Surg Br. 2003;85(2):231-237. PMID: 12678358. The PubMed page clearly shows the title, authors, and PMID in this way. A Giannoudis record more relevant to damage-control orthopedics is: Giannoudis PV, Pape HC. Damage control orthopaedics in unstable pelvic ring injuries. Injury. 2004;35(7):671-677. PMID: 15203307. Search results indicate that this article addresses the damage-control orthopedics approach in unstable/in extremis multitrauma patients.	0/1. Content relevance could not be assessed because the reference was not bibliographically verifiable.
10.3	Halawi MJ, Morwood MP. Acute Management of Open Fractures: An Evidence-Based Review. Orthopedics. 2015;38(11):e1025-e1033. doi:10.3928/01477447-20151020-12. PMID: 26558667.	2/2. The reference was verified previously: Halawi MJ, Morwood MP. Acute Management of Open Fractures: An Evidence-Based Review. Orthopedics. 2015;38(11):e1025-e1033. doi:10.3928/01477447-20151020-12. PMID: 26558667.	1/1. The article discusses the acute management of open fractures through key steps such as antibiotic therapy, irrigation and debridement, fracture stabilization, soft tissue management, and complication follow-up. Therefore, it can be used as a source supporting general open fracture management for the “fracture stabilization” heading. It is directly related to acute open fracture management. However, it is not the most specific source by itself for the stabilization subheading; it should preferably be used together with an article on open tibia fracture fixation or damage-control fixation.
11.1	Godina M. Early microsurgical reconstruction of complex trauma of the extremities. Plast Reconstr Surg. PMID: 3889367	1/2. The provided title and author are correct, but the PMID is incorrect. Correct PubMed record: Godina M. Early microsurgical reconstruction of complex trauma of the extremities. Plast Reconstr Surg. 1986;78(3):285-292. PMID: 3737751. The provided PMID 3889367 does not match this article. In the search, the correct record appears as PMID 3737751.	1/1. The article evaluated the outcomes of microsurgical reconstruction in 532 patients with extremity trauma; patients were grouped according to the timing of reconstruction. Better outcomes were reported in the early reconstruction group. This study is a classic reference for early soft tissue reconstruction/flap timing in extremity trauma. It is appropriate for supporting the principle of early flap/soft tissue coverage in severe open fractures.
11.2	Gopal S, et al. Fix and flap concept in severe open tibial fractures. J Bone Joint Surg Br. PMID: 11132261	1/2. The topic of the provided reference is correct, but the PMID is incorrect. Correct PubMed record: Gopal S, Majumder S, Batchelor AGB, Knight SL, De Boer P, Smith RM. Fix and flap: the radical orthopaedic and plastic treatment of severe open fractures of the tibia. J Bone Joint Surg Br. 2000;82(7):959-966. PMID: 11041582. The PubMed record gives this article with PMID 11041582.	1/1. The article retrospectively evaluated 84 patients with severe Gustilo IIIB/IIIC open tibia fractures and states that early soft tissue coverage and stabilization with a combined orthopedic and plastic surgery approach produced good results. The “fix and flap” concept is directly appropriate for the stabilization + early flap coverage heading in severe open tibia fractures.
11.3	Pollak AN, et al. Delay in flap coverage increases complications. J Bone Joint Surg Am. PMID: 15118029	0/2. The provided PMID is completely incorrect. No article with the same title was found. In PubMed, PMID 15118029 does not belong to Pollak or to an article on delayed flap coverage in open tibia fractures; it belongs to the following article: King PJ, Malin AS, Scott RD, Thornhill TS. The fate of patients not returning for follow-up five years after total knee arthroplasty. J Bone Joint Surg Am. 2004;86(5):897-901. PMID: 15118029.	0/1. Content relevance could not be assessed because the reference was not bibliographically verifiable.
12.1	Costa ML, et al. WOLLF trial: NPWT vs standard dressings in severe open fractures. BMJ. PMID: 29896626	1/2. The provided study is correct and the PMID is correct, but the journal information is incorrect. Correct PubMed record: Costa ML, et al. Effect of Negative Pressure Wound Therapy vs Standard Wound Management on 12-Month Disability Among Adults With Severe Open Fracture of the Lower Limb: The WOLLF Randomized Clinical Trial. JAMA. 2018;319(22):2280-2288. PMID: 29896626. Therefore, the study is not a BMJ article, but a JAMA article.	1/1. The article compares negative pressure wound therapy (NPWT) with standard wound management after initial surgical debridement in severe open lower extremity fractures in terms of 12-month disability, deep infection, and quality of life. It is directly appropriate for comparing NPWT with standard dressings in open fractures.
12.2	Stannard JP, et al. NPWT after severe open fractures. J Orthop Trauma. PMID: 19318864	1/2. The provided study is real and the topic is correct, but the PMID is incorrect. Correct PubMed record: Stannard JP, Volgas DA, McGwin G 3rd, Stewart RL, Obremskey W, Moore T, Anglen JO. Negative pressure wound therapy after severe open fractures: a prospective randomized study. J Orthop Trauma. 2009;23(8):552-557. PMID: 19704269. The provided PMID 19318864 does not match this article.	1/1. The article evaluates the effect of negative pressure wound therapy after severe open fractures on deep infection rates in a prospective randomized design. In this respect, it is an appropriate source supporting the relationship between NPWT use and infection development in high-energy open fractures. It is directly appropriate for NPWT use in open fractures.
12.3	Blum ML, et al. NPWT systematic review in open tibia fractures. Injury. PMID: 22840312	1/2. The provided reference is incorrect. PMID 22840312 does not appear to match this article. The correct PubMed record is as follows: Blum ML, Esser M, Richardson M, Paul E, Rosenfeldt FL. Negative pressure wound therapy reduces deep infection rate in open tibial fractures. J Orthop Trauma. 2012;26(9):499-505. PMID: 22487900. In addition, the provided statement says “systematic review”; however, the correct Blum article is not a systematic review, but a clinical study evaluating NPWT and the deep infection rate in open tibia fractures.	1/1. The article reports findings suggesting that NPWT reduces the deep infection rate when used as a dressing for traumatic wounds in open tibial fractures. It is appropriate for the NPWT and deep infection heading in open tibia fractures. However, describing the reference as a “systematic review” is incorrect.
13.1	Fox N, et al. Evaluation and management of penetrating/extremity vascular trauma. J Trauma Acute Care Surg. PMID: 29319660	1/2. The topic of the provided reference is close to correct, but the PMID is incorrect. Correct PubMed record: Fox N, Rajani RR, Bokhari F, Chiu WC, Kerwin A, Seamon MJ, et al. Evaluation and management of penetrating lower extremity arterial trauma: an Eastern Association for the Surgery of Trauma practice management guideline. J Trauma Acute Care Surg. 2012;73(5 Suppl 4):S315-S320. PMID: 23114487. The provided PMID 29319660 does not match this study.	1/1. The article is an EAST practice management guideline addressing the assessment, diagnosis, and treatment approach in penetrating lower extremity arterial trauma. Therefore, it can be used to support arterial evaluation, diagnostic algorithms, and treatment planning in vascular injuries accompanying open fractures. It is appropriate for the assessment of concomitant vascular injury / ischemia / arterial trauma in open fractures. It is not specific to open fractures, but it supports the vascular injury heading.
13.2	Helfet DL, et al. Limb salvage after vascular injury with fractures. Clin Orthop Relat Res. PMID: 7634671	0/2. I could not verify this reference with the provided PMID. A reliable PubMed match for Helfet DL / limb salvage / vascular injury with fractures / PMID 7634671 did not appear in the search. One PubMed record found close to this topic is: Al-Salman MM, Al-Khawashki H, Sindig K, Rabee H, Abu-Zidan FM. Vascular injuries associated with limb fractures. Injury. 1997;28(2):103-107. PMID: 9205574. However, this is not the provided Helfet reference.	0/1. Content relevance could not be assessed because the reference was not bibliographically verifiable.
13.3	Johansen K, et al. Mangled extremity severity score. J Trauma. PMID: 2342140	2/2. The provided PMID belongs to the correct article. Correct PubMed record: Johansen K, Daines M, Howey T, Helfet D, Hansen ST Jr. Objective criteria accurately predict amputation following lower extremity trauma. J Trauma. 1990;30(5):568-572. PMID: 2342140. The title was abbreviated as “Mangled extremity severity score”; the full title is as above. Despite this minor deficiency, the PMID/author/topic match is correct, so we assign 2/2 for bibliographic accuracy.	1/1. The article states that the MESS is a simple score for lower extremity trauma based on skeletal/soft tissue injury, extremity ischemia, shock, and age criteria, and that it is used to predict the need for amputation. It is appropriate for severe vascular injury / mangled extremity / limb salvage-amputation decisions accompanying open fractures. It does not provide vascular injury management directly in the form of a treatment algorithm; however, it is a classic reference supporting prognosis and amputation decisions in severe extremity trauma.
14.1	Olson SA, Glasgow RR. Acute compartment syndrome in lower extremity trauma. J Am Acad Orthop Surg. PMID: 16467184	1/2. The provided reference is close to correct, but the PMID is incorrect. Correct PubMed record: Olson SA, Glasgow RR. Acute compartment syndrome in lower extremity musculoskeletal trauma. J Am Acad Orthop Surg. 2005;13(7):436-444. PMID: 16272268. The provided PMID 16467184 does not match this article. In PubMed, the correct PMID appears to be 16272268.	1/1. The article states that acute compartment syndrome impairs tissue perfusion because of increased pressure within an osteofascial compartment; it is described as a serious and potentially devastating complication in lower extremity trauma. The importance of diagnosis and early treatment is emphasized. It is appropriate for compartment syndrome surveillance after open fractures. It is not specific to open fractures, but it supports compartment syndrome associated with lower extremity trauma and fractures.
14.2	McQueen MM, Duckworth AD. Diagnosis and management of compartment syndrome. Bone Joint J. PMID: 24395314	0/2. I could not verify the provided reference in its current form. No match was found for PMID 24395314 with “McQueen MM, Duckworth AD. Diagnosis and management of compartment syndrome. Bone Joint J.” The closer PubMed records are as follows: - McQueen MM, Duckworth AD. The diagnosis of acute compartment syndrome: a review. Eur J Trauma Emerg Surg. 2014. PMID: 26814506. - Duckworth AD, McQueen MM. The Diagnosis of Acute Compartment Syndrome: A Critical Analysis Review. JBJS Rev. 2017. PMID: 29206684.	0/1. Content relevance could not be assessed because the reference was not bibliographically verifiable.
14.3	Matsen FA III. Compartmental syndromes. N Engl J Med. PMID: 7466320	1/2. The provided reference is incorrect. There are NEJM publications by Matsen titled “Compartmental syndromes” in PubMed; however, the provided PMID 7466320 does not appear to be correct. PubMed records close to the correct reference: 1. Matsen FA 3rd. Compartmental syndromes. N Engl J Med. 1979;300(21):1210-1211. PMID: 431649. 2. Matsen FA 3rd. Compartmental syndromes. Hosp Pract. 1980. PMID: 7409781.	1/1. In the closest articles, compartment syndrome is described as a condition in which increased compartment pressure impairs circulation; it is stated that it should be suspected in cases of pain/neuromuscular deficit and can be confirmed by increased pressure.
15.1	Metsemakers WJ, et al. Fracture-related infection definition and management. Injury. PMID: 28843446	1/2. The provided reference is close to correct in topic, but the PMID is incorrect. Correct PubMed record: Metsemakers WJ, Morgenstern M, McNally MA, Moriarty TF, McFadyen I, Scarborough M, et al. Fracture-related infection: A consensus on definition from an international expert group. Injury. 2018;49(3):505-510. PMID: 28867644. The provided PMID 28843446 does not match this article.	1/1. The article states that fracture-related infection is a common and serious complication in trauma surgery; that the lack of a clear definition makes comparison between studies difficult; and that, therefore, an international expert group developed a consensus definition for FRI. It is appropriate for the definition of infection/FRI and complication surveillance after open fracture. The term “Management” is too broad in the title; this article is mainly a definition/diagnostic framework source.
15.2	Zimmerli W. Clinical practice: bone and joint infections. N Engl J Med. PMID: 16554528	0/2. The provided reference is incorrect. PMID 16554528 does not belong to Zimmerli’s bone and joint infection article, but to a NEJM article on LDL/PCSK9 and coronary events. Zimmerli’s relevant and well-known article in NEJM is as follows: Zimmerli W, Trampuz A, Ochsner PE. Prosthetic-joint infections. N Engl J Med. 2004;351(16):1645-1654. PMID: 15483283. However, this article is also about prosthetic joint infection; it is not a directly ideal source for open fracture infection or fracture-related infection.	0/1. Content relevance could not be assessed because the reference was not bibliographically verifiable.
15.3	Court-Brown CM, et al. Open fracture outcomes and complications. Injury. PMID: 11311731	0/2. This reference is incorrect. As seen previously, PMID 11311731 is not related to Court-Brown or open fracture complications; it is an article on neuropeptide Y/naloxone. Court-Brown’s actual PubMed record related to open fractures is as follows: Court-Brown CM, Rimmer S, Prakash U, McQueen MM. The epidemiology of open long bone fractures. Injury. 1998;29(7):529-534. PMID: 10193496. The provided PMID 11311731 is not related to open fractures. In the abstract of the correct Court-Brown article, PMID 10193496, the incidence, age-sex distribution, and epidemiology of open long-bone fractures are addressed. However, this correct Court-Brown article is also primarily an epidemiology source; it is not the strongest reference for complication/outcome management. The content of the provided PMID is not related to open fracture complications.	0/1. Content relevance could not be assessed because the reference was not bibliographically verifiable.

Appendix E

**Table 8:** Independent reference-level scores assigned by Reviewer 1 (n = 45)

No	Reference	Bibliographic accuracy	Content relevance
1.1	ACS / ATLS principles	0/2	0/1
1.2	Giannoudis PV et al. Damage control orthopaedics / PMID 12678358	0/2	0/1
1.3	Pape HC et al. Timing of fracture fixation / PMID 15712904	1/2	1/1
2.1	Halawi MJ, Morwood MP. Acute Management of Open Fractures / PMID 26558667	2/2	1/1
2.2	Rockwood and Green’s Fractures in Adults	0/2	0/1
2.3	Brinker MR, O’Connor DP. Limb-threatening extremity trauma / PMID 19385542	0/2	0/1
3.1	Halawi MJ, Morwood MP / PMID 26558667	2/2	1/1
3.2	Pollak AN et al. Temporary stabilization / PMID 12828449	0/2	0/1
3.3	Court-Brown CM et al. Initial management principles / PMID 11311731	0/2	0/1
4.1	Patzakis MJ, Wilkins J. Factors influencing infection rate / PMID 2721073	2/2	1/1
4.2	Lack WD et al. Type III open tibia fractures / PMID 25581616	1/2	1/1
4.3	Halawi MJ, Morwood MP / PMID 26558667	2/2	1/1
5.1	Hoff WS et al. Antibiotic guideline / PMID 15094570	1/2	1/1
5.2	Hauser CJ et al. Antibiotic management / PMID 19673668	1/2	1/1
5.3	Halawi MJ, Morwood MP / PMID 26558667	2/2	1/1
6.1	CDC tetanus wound management recommendations	0/2	0/1
6.2	Edlich RF et al. Management and prevention of tetanus / PMID 11883546	1/2	1/1
6.3	Halawi MJ, Morwood MP / PMID 26558667	2/2	1/1
7.1	Halawi MJ, Morwood MP / PMID 26558667	2/2	1/1
7.2	Tornetta P III et al. CT role in periarticular fracture planning / PMID 15213497	0/2	0/1
7.3	Inaba K et al. CTA for extremity vascular trauma / PMID 18376169	0/2	0/1
8.1	Gustilo RB, Anderson JT. Prevention of infection / PMID 773941	2/2	1/1
8.2	Gustilo RB et al. Type III open fractures / PMID 6708156	1/2	1/1
8.3	Kim PH, Leopold SS. Gustilo-Anderson classification review / PMID 22207507	1/2	1/1
9.1	FLOW Investigators. Wound irrigation / PMID 26465983	1/2	1/1
9.2	Anglen JO. Wound irrigation in musculoskeletal injury / PMID 11281638	1/2	1/1
9.3	Halawi MJ, Morwood MP / PMID 26558667	2/2	1/1
10.1	Bhandari M et al. Reamed vs unreamed nailing / PMID 16322610	1/2	1/1
10.2	Giannoudis PV et al. External fixation in DCO / PMID 12678358	0/2	0/1
10.3	Halawi MJ, Morwood MP / PMID 26558667	2/2	1/1
11.1	Godina M. Early microsurgical reconstruction / PMID 3889367	1/2	1/1
11.2	Gopal S et al. Fix and flap / PMID 11132261	1/2	1/1
11.3	Pollak AN et al. Delay in flap coverage / PMID 15118029	0/2	0/1
12.1	Costa ML et al. WOLLF trial / PMID 29896626	1/2	1/1
12.2	Stannard JP et al. NPWT / PMID 19318864	1/2	1/1
12.3	Blum ML et al. NPWT systematic review / PMID 22840312	1/2	1/1
13.1	Fox N et al. Extremity vascular trauma / PMID 29319660	1/2	1/1
13.2	Helfet DL et al. Limb salvage after vascular injury / PMID 7634671	0/2	0/1
13.3	Johansen K et al. Mangled extremity severity score / PMID 2342140	2/2	1/1
14.1	Olson SA, Glasgow RR. Acute compartment syndrome / PMID 16467184	1/2	1/1
14.2	McQueen MM, Duckworth AD. Diagnosis and management of compartment syndrome / PMID 24395314	0/2	0/1
14.3	Matsen FA III. Compartmental syndromes / PMID 7466320	1/2	1/1
15.1	Metsemakers WJ et al. Fracture-related infection / PMID 28843446	1/2	1/1
15.2	Zimmerli W. Bone and joint infections / PMID 16554528	0/2	0/1
15.3	Court-Brown CM et al. Open fracture outcomes / PMID 11311731	0/2	0/1

Appendix F

**Table 9:** Independent reference-level scores assigned by Reviewer 2 (n = 45)

No	Reference	Bibliographic accuracy	Content relevance
1.1	ACS / ATLS principles	0/2	0/1
1.2	Giannoudis PV et al. Damage control orthopaedics / PMID 12678358	0/2	0/1
1.3	Pape HC et al. Timing of fracture fixation / PMID 15712904	1/2	1/1
2.1	Halawi MJ, Morwood MP. Acute Management of Open Fractures / PMID 26558667	2/2	1/1
2.2	Rockwood and Green’s Fractures in Adults	0/2	0/1
2.3	Brinker MR, O’Connor DP. Limb-threatening extremity trauma / PMID 19385542	0/2	0/1
3.1	Halawi MJ, Morwood MP / PMID 26558667	2/2	1/1
3.2	Pollak AN et al. Temporary stabilization / PMID 12828449	0/2	0/1
3.3	Court-Brown CM et al. Initial management principles / PMID 11311731	0/2	0/1
4.1	Patzakis MJ, Wilkins J. Factors influencing infection rate / PMID 2721073	2/2	1/1
4.2	Lack WD et al. Type III open tibia fractures / PMID 25581616	1/2	1/1
4.3	Halawi MJ, Morwood MP / PMID 26558667	2/2	1/1
5.1	Hoff WS et al. Antibiotic guideline / PMID 15094570	1/2	1/1
5.2	Hauser CJ et al. Antibiotic management / PMID 19673668	0/2	0/1
5.3	Halawi MJ, Morwood MP / PMID 26558667	2/2	1/1
6.1	CDC tetanus wound management recommendations	0/2	0/1
6.2	Edlich RF et al. Management and prevention of tetanus / PMID 11883546	1/2	1/1
6.3	Halawi MJ, Morwood MP / PMID 26558667	2/2	1/1
7.1	Halawi MJ, Morwood MP / PMID 26558667	2/2	1/1
7.2	Tornetta P III et al. CT role in periarticular fracture planning / PMID 15213497	0/2	0/1
7.3	Inaba K et al. CTA for extremity vascular trauma / PMID 18376169	0/2	0/1
8.1	Gustilo RB, Anderson JT. Prevention of infection / PMID 773941	2/2	1/1
8.2	Gustilo RB et al. Type III open fractures / PMID 6708156	1/2	1/1
8.3	Kim PH, Leopold SS. Gustilo-Anderson classification review / PMID 22207507	1/2	1/1
9.1	FLOW Investigators. Wound irrigation / PMID 26465983	1/2	1/1
9.2	Anglen JO. Wound irrigation in musculoskeletal injury / PMID 11281638	1/2	1/1
9.3	Halawi MJ, Morwood MP / PMID 26558667	2/2	1/1
10.1	Bhandari M et al. Reamed vs unreamed nailing / PMID 16322610	1/2	1/1
10.2	Giannoudis PV et al. External fixation in DCO / PMID 12678358	0/2	0/1
10.3	Halawi MJ, Morwood MP / PMID 26558667	2/2	1/1
11.1	Godina M. Early microsurgical reconstruction / PMID 3889367	1/2	1/1
11.2	Gopal S et al. Fix and flap / PMID 11132261	1/2	1/1
11.3	Pollak AN et al. Delay in flap coverage / PMID 15118029	0/2	0/1
12.1	Costa ML et al. WOLLF trial / PMID 29896626	1/2	1/1
12.2	Stannard JP et al. NPWT / PMID 19318864	1/2	1/1
12.3	Blum ML et al. NPWT systematic review / PMID 22840312	1/2	1/1
13.1	Fox N et al. Extremity vascular trauma / PMID 29319660	1/2	1/1
13.2	Helfet DL et al. Limb salvage after vascular injury / PMID 7634671	0/2	0/1
13.3	Johansen K et al. Mangled extremity severity score / PMID 2342140	2/2	1/1
14.1	Olson SA, Glasgow RR. Acute compartment syndrome / PMID 16467184	1/2	1/1
14.2	McQueen MM, Duckworth AD. Diagnosis and management of compartment syndrome / PMID 24395314	0/2	0/1
14.3	Matsen FA III. Compartmental syndromes / PMID 7466320	1/2	1/1
15.1	Metsemakers WJ et al. Fracture-related infection / PMID 28843446	1/2	1/1
15.2	Zimmerli W. Bone and joint infections / PMID 16554528	0/2	0/1
15.3	Court-Brown CM et al. Open fracture outcomes / PMID 11311731	0/2	0/1

Appendix G

**Table 10:** Final consensus reference-level scores (n = 45)

No	Reference	Bibliographic accuracy	Content relevance
1.1	ACS / ATLS principles	0/2	0/1
1.2	Giannoudis PV et al. Damage control orthopaedics / PMID 12678358	0/2	0/1
1.3	Pape HC et al. Timing of fracture fixation / PMID 15712904	1/2	1/1
2.1	Halawi MJ, Morwood MP. Acute Management of Open Fractures / PMID 26558667	2/2	1/1
2.2	Rockwood and Green’s Fractures in Adults	0/2	0/1
2.3	Brinker MR, O’Connor DP. Limb-threatening extremity trauma / PMID 19385542	0/2	0/1
3.1	Halawi MJ, Morwood MP / PMID 26558667	2/2	1/1
3.2	Pollak AN et al. Temporary stabilization / PMID 12828449	0/2	0/1
3.3	Court-Brown CM et al. Initial management principles / PMID 11311731	0/2	0/1
4.1	Patzakis MJ, Wilkins J. Factors influencing infection rate / PMID 2721073	2/2	1/1
4.2	Lack WD et al. Type III open tibia fractures / PMID 25581616	1/2	1/1
4.3	Halawi MJ, Morwood MP / PMID 26558667	2/2	1/1
5.1	Hoff WS et al. Antibiotic guideline / PMID 15094570	1/2	1/1
5.2	Hauser CJ et al. Antibiotic management / PMID 19673668	1/2	1/1
5.3	Halawi MJ, Morwood MP / PMID 26558667	2/2	1/1
6.1	CDC tetanus wound management recommendations	0/2	0/1
6.2	Edlich RF et al. Management and prevention of tetanus / PMID 11883546	1/2	1/1
6.3	Halawi MJ, Morwood MP / PMID 26558667	2/2	1/1
7.1	Halawi MJ, Morwood MP / PMID 26558667	2/2	1/1
7.2	Tornetta P III et al. CT role in periarticular fracture planning / PMID 15213497	0/2	0/1
7.3	Inaba K et al. CTA for extremity vascular trauma / PMID 18376169	0/2	0/1
8.1	Gustilo RB, Anderson JT. Prevention of infection / PMID 773941	2/2	1/1
8.2	Gustilo RB et al. Type III open fractures / PMID 6708156	1/2	1/1
8.3	Kim PH, Leopold SS. Gustilo-Anderson classification review / PMID 22207507	1/2	1/1
9.1	FLOW Investigators. Wound irrigation / PMID 26465983	1/2	1/1
9.2	Anglen JO. Wound irrigation in musculoskeletal injury / PMID 11281638	1/2	1/1
9.3	Halawi MJ, Morwood MP / PMID 26558667	2/2	1/1
10.1	Bhandari M et al. Reamed vs unreamed nailing / PMID 16322610	1/2	1/1
10.2	Giannoudis PV et al. External fixation in DCO / PMID 12678358	0/2	0/1
10.3	Halawi MJ, Morwood MP / PMID 26558667	2/2	1/1
11.1	Godina M. Early microsurgical reconstruction / PMID 3889367	1/2	1/1
11.2	Gopal S et al. Fix and flap / PMID 11132261	1/2	1/1
11.3	Pollak AN et al. Delay in flap coverage / PMID 15118029	0/2	0/1
12.1	Costa ML et al. WOLLF trial / PMID 29896626	1/2	1/1
12.2	Stannard JP et al. NPWT / PMID 19318864	1/2	1/1
12.3	Blum ML et al. NPWT systematic review / PMID 22840312	1/2	1/1
13.1	Fox N et al. Extremity vascular trauma / PMID 29319660	1/2	1/1
13.2	Helfet DL et al. Limb salvage after vascular injury / PMID 7634671	0/2	0/1
13.3	Johansen K et al. Mangled extremity severity score / PMID 2342140	2/2	1/1
14.1	Olson SA, Glasgow RR. Acute compartment syndrome / PMID 16467184	1/2	1/1
14.2	McQueen MM, Duckworth AD. Diagnosis and management of compartment syndrome / PMID 24395314	0/2	0/1
14.3	Matsen FA III. Compartmental syndromes / PMID 7466320	1/2	1/1
15.1	Metsemakers WJ et al. Fracture-related infection / PMID 28843446	1/2	1/1
15.2	Zimmerli W. Bone and joint infections / PMID 16554528	0/2	0/1
15.3	Court-Brown CM et al. Open fracture outcomes / PMID 11311731	0/2	0/1
